# Social Network Size and Subjective Well-Being: The Mediating Role of Future Time Perspective Among Community-Dwelling Retirees

**DOI:** 10.3389/fpsyg.2019.02590

**Published:** 2019-11-15

**Authors:** Zhen Zhang, Jianxin Zhang, Na Zhao, Yang Yang

**Affiliations:** ^1^Key Laboratory of Behavioral Sciences, Institute of Psychology, Chinese Academy of Sciences, Beijing, China; ^2^Department of Psychology, University of Chinese Academy of Sciences, Beijing, China; ^3^Key Laboratory of Mental Health, Institute of Psychology, Chinese Academy of Sciences, Beijing, China; ^4^School of Sociology and Psychology, Central University of Finance and Economics, Beijing, China; ^5^Department of Psychology, Cangzhou Medical College, Cangzhou, China

**Keywords:** social network size, future time perspective, subjective well-being, community-dwelling retirees, life satisfaction, positive and negative affect, meaning in life

## Abstract

An accumulating body of literature has confirmed the effect of social networks on the subjective well-being (SWB). However, the relevant mechanism for the relationship between them requires further exploration. This research examined the association between social network size and SWB and the mediating role of future time perspective (FTP) among Chinese retirees. We modeled the relationship between social network size, FTP, and SWB by two sub-studies. SWB was indicated by life satisfaction, positive affect, negative affect, and meaning in life. FTP comprised two dimensions: focusing on opportunity in future and focusing on limitation of time. Study 1 used the number of Spring Festival greeters, and Study 2 used the size of networks based on common actions (discussion, mutual helping, and social participation) as indicators of network size to examine the association and mediating effect among 1097 and 335 community-dwelling retirees, respectively. Both studies revealed that social network size was positively associated with SWB; FTP-opportunity but not FTP-limitation mediated above associations, when possible confounding variables were controlled for. Findings confirm relevance of social networks in the SWB of retirees, and provide a new insight into the role of FTP as an explanatory mechanism.

## Introduction

Previous studies have generally confirmed that social networks are powerful determinants of subjective well-being (SWB) of elderly (Pinquart and Sörensen, 2000; Huxhold et al., 2013). The size of a social network affected or were related to the well-being and quality of life of older adults (Rafnsson et al., 2015; Wang, 2016; Moorman and Boerner, 2017; Schwartz and Litwin, 2019). Rafnsson et al. (2015) revealed that social network size, but not social network diversity, was positively associated with future life satisfaction and quality of life of older adults. Nevertheless, there are relatively few empirical studies exploring the mechanisms that explain why SWB can benefit from a large social network (Huxhold et al., 2013; Fuller-Iglesias, 2015; Webster et al., 2015). In recent years, time perspective, especially future time perspective (FTP), has been considered as a vital factor affecting one’s mental health and well-being (Sailer et al., 2014; Webster et al., 2014; Hoppmann et al., 2017). Correlational and causal relationships between social networks and time perspective have been confirmed, to some extent (Fung et al., 2001; Lang and Carstensen, 2002; Holman and Zimbardo, 2009). However, up to now, few empirical studies examine the relational mechanisms among social network, time perspective, and SWB, simultaneously. Based on the extant theories and literature on social networks, time perspective, and the SWB, we speculate that an association may exist in shaping and maintaining SWB. Size of social network can directly relate to or influence the SWB of older adults. Furthermore, size of social network can render one’s FTP more expansive and less limited, subsequently improving SWB of older adults.

### Social Networks and FTP

Future time perspective is a psychological construal of the remaining time in one’s perceived life span and should be linked with, but not equal to, chronological age (Lang and Carstensen, 2002; Carstensen, 2006; Grühn et al., 2016). FTP is defined from positive (extension and opportunity) and negative aspects (limitations and constraints). A higher FTP means one feels having more time and opportunity and fewer constraints to pursue diverse social goals (Rohr et al., 2017). Having a large or supportive social network was associated with an expansive FTP of older adults (Lang and Carstensen, 2002; Windsor et al., 2012). Carstensen (2006) concludes that with increasing age, personal goals and motivations can change to select smaller and more intimate networks such as family members and close friends. This means that a limited FTP is related to a small social network and an expansive FTP to a larger or more diverse social network. Recent empirical studies based on questionnaire measures (Lang and Carstensen, 2002; Cate and John, 2007) and experimental manipulation (Cheng and Yim, 2008) confirmed a positive relationship between FTP and social network. However, a small number of extant literatures seem put more emphasis on effects of FTP on social network rather than opposite direction. Very few studies consider social network as predictors of FTP.

### FTP and SWB

The perception of future time, including subjective nearness to death and future life expectancy, is vital in aging research, because of its implications for SWB. Recent studies confirmed a connection between how people perceive their past, present, and future and their SWB and health-promoting behaviors (Kotter-Grühn and Smith, 2011; Stahl and Patrick, 2012; Sailer et al., 2014; Grühn et al., 2016; Hoppmann et al., 2017). A recent meta-analysis article concluded that individuals higher on FTP reported higher levels of life satisfaction and subjective health, and lower levels of anxiety and depression (Kooij et al., 2018). Sailer et al. (2014) related having FTP or planning for the future to psychological well-being and positive affect. Hoppmann et al. (2017) found that limited FTP was related to less life satisfaction and positive affect, as well as to higher negative affect and depressive symptoms. Kotter-Grühn and Smith (2011) found that older adults who reported lower levels of future orientation demonstrated decreased well-being over time. Several studies also showed that FTP as a subjective sense is more closely related to SWB than chronological age (Allemand et al., 2012; Demiray and Bluck, 2014). Overall, multiple empirical studies supported the positive effect of expansive rather limited FTP on SWB.

Although recent studies indicated that FTP was positively related to or beneficial for SWB, some inferences and empirical findings show a negative relationship between them (Carstensen, 2006; Yeung et al., 2007). The basic inference of socio-emotional selectivity theory (SST) is that when time is perceived as limited, not expansive, motivations and goals related to emotional meaning are prioritized over future-oriented motivations and goals to acquire information (Lockehoff and Carstensen, 2004). Similarly, it can be inferred that older adults with a limited FTP seek more emotional satisfaction and further experience or maintain a higher SWB than those with an expansive FTP (Lang and Carstensen, 2002; Carstensen, 2006). Yeung et al. (2007) found that women with a more limited FTP were happier when they had fewer close friends in their social networks. In view of this ambiguity in the relationship between FTP and SWB based on the extant literature, the relationship valence between FTP and SWB needs further confirmation.

### Mediating Role of FTP Between Social Network and SWB

In addition to the direct relationship between social network and SWB, FTP may indirectly account for a connection between social network and SWB. According to the accessibility of social resources theory, although social networks do not automatically yield social resources, they are main channels for the exchange and flow of social resources (House et al., 1988; Lin, 2001). First, a large social network often means more social support and increased access to resources, which provides individuals with more opportunities to finish tasks, as well as the perception of fewer constraints and barriers in the future (Rohr et al., 2017). Thus, we speculate that a larger social network subjectively extends one’s perception of remaining time and enhances one’s hope for future life. With an equal chronological age, extensive social relationship networks may extend the FTP for older adults. Furthermore, large social networks often signify diversity in social relationships and interactions. Relative to restricted networks, a diverse social network provides diverse sources of acquiring information. That is, if one can readily access more information, one will have a more expansive FTP. Carstensen and colleagues confirmed the positive relatedness of FTP and seeking information among older adults (Lang and Carstensen, 2002; Carstensen, 2006). For example, by virtue of a large or diverse network, older adults can acquire more new knowledge on preventative and health-promoting measures (Shiovitz-Ezra and Litwin, 2012), which in turn, may promote their SWB. Similar with view of social resource theory, social integration theory thought that network size is an important indicator of social integration. A bigger social network size often means a higher degree of social integration and lower degree of social isolation, which determines one’s opportunity for access to social capital as resources embedded in social network (Song, 2011). That is to say, perception of more opportunities and less limitations in future may bridge the relationship between social network and SWB.

Reaching retirement is a significant life stage (Shultz and Wang, 2011). On the one hand, retirement often means approaching or reaching old age. Retirees feel that their remaining time is more limited, while their original social network is gradually shrinking. On the other hand, some researchers noted that for some retirees, a focus on future plans and opportunities may increase. They often have more time to do what they are interested in such as leisure activities, entertainment, and participation in social activities (Moen and Flood, 2013). These activities are associated with a more positive life attitude and happiness. Thus, individual differences in social networks and the FTP of retirees may be an important source of SWB. In this study, using retired community residents as the sample, we attempted to confirm whether large social networks can enhance retirees’ SWB in multiple aspects through expanding their FTP. The present study consisted of two sub-studies that used the number of Spring Festival greeters and size of important relationship networks were employed as indicators of social network size, respectively.

In addition, in this study, SWB was represented by four indicators: life satisfaction, positive affect, negative affect, and meaning in life. High life satisfaction, high positive affect, and low negative affect are three core components of SWB or happiness (Diener et al., 1985). A dual-orientation approach groups the above three indicators as pertaining to the hedonic aspect of SWB, which emphasizes maximizing pleasure and minimizing pain for a good life; meaning in life pertains to the eudaimonic aspect of SWB, which emphasizes the importance of seeking a worthy and meaningful life (McMahan and Estes, 2011). The researchers believe that a complete understanding of SWB should cover both hedonic and eudaimonic aspects.

### Present Study

We aimed to explore the association between social network size and SWB and mediating role of FTP among retired middle-aged and elderly people. We endeavored to confirm that large social networks are positively related to retirees’ SWB in multiple aspects through expanding their FTP. Assumptions were as follows:

Hypothesis 1: Social network size is positively correlated with the SWB of Chinese retirees. That is, retirees with a larger social network size often have higher SWB.
Hypothesis 2: Social network size is positively correlated with FTP. According to empirical studies, FTP consists of two relatively independent factors: focusing on future opportunities (FTP-opportunity) and focusing on the limitation of time (FTP-limitation) (Cate and John, 2007). These two factors can represent unique meaning in perception of the future (Cate and John, 2007; Kozik et al., 2015). This means that retirees with a larger social network size are often more focused on opportunities in the future and less focused on limited time.
Hypothesis 3: FTP (FTP-opportunity and FTP-limitation) as a subjective sense of time is positively correlated with SWB. Specifically, retirees with higher FTP-opportunity and lower FTP-limitation often have higher SWB.
Hypothesis 4: FTP (FTP-opportunity and FTP-limitation) mediates the relationship between social network size and SWB, after controlling for demographic and socio-economic variables. Thus, we hypothesize that retirees with a larger social network size are often more focused on opportunities in the future and less focus on limited time, which results in higher SWB.

## Study 1: Size of Spring Festival Greeting Network as the Independent Variable

In Study 1, the number of Spring Festival greeters was used as the indicator of social network size to examine the relationship between social network size and SWB and the mediating role of FTP. The data from Studies 1 and 2 are part of a research project examining the social organizations and quality of life of middle-aged and elderly people in China.

### Participants and Procedure

Participants provided written informed consent after the investigators explained the aim and requirements before conducting the investigation. Participation in the study was anonymous and individual-based. The investigators were psychological researchers, psychology graduate students, and service workers from local community committees. The academic and ethics committees of the corresponding authors’ affiliated unit approved this study.

Using a stratified sampling method, retirees were recruited from 35 neighborhoods of a medium-sized city in terms of economic development and population in China. In China, the minimum retirement age is often 50 or 55 years for women and 60 for men; thus, we selected retired community-dwelling residents aged 50 years or older as participants. Of the 1,200 questionnaires distributed, 1,097 were returned. Of the returned questionnaires, 29 were invalid because of high incompleteness (20% missing rate or more). Finally, the valid sample consisted of 1,068 participants (valid response rate = 89%): 422 men, 644 women, and 2 participants who did not report their sex. Their ages ranged from 50 to 92 years (see Table 1).

**TABLE 1:** Demographic and socio-economic characteristics of participants in Study 1.

**Variable**	***n***	**Valid%**	**Mean (*SD*)**
**Sex**			
Male	422	39.6	
Female	644	60.4	
Missing	2		
**Age**			62.75 (8.03)
59 and younger	384	36.4	
60–69 years	458	43.5	
70–79 years	173	16.4	
80 and older	39	3.7	
Missing	14		
**Educational level**			
Elementary or lower	157	15.3	
Junior high school	426	41.6	
Senior high school	343	33.5	
Junior college or higher	98	9.6	
Missing	44		
**Marital status**			
Single(Widowed/unmarried/divorced/separated)	150	14.4	
With a spouse	895	85.6	
Missing	23		
**Household yearly income (RMB)**			
Less than 20000	172	16.3	
20000–30000	293	27.8	
30000–50000	321	30.5	
50000-80000	184	17.5	
80000–120000	75	7.1	
More than 120000	9	0.9	
Missing	23		

The investigation was conducted from June to August 2017. Participants completed the questionnaires independently in their homes or a public place (such as community meeting room). Each participant took approximately 30 min to complete this investigation. Once completed, each participant was paid RMB 40 (about $6) as compensation.

### Measures

#### Social Network Size

Based on indigenized considerations, we adopted the number of an individual’s Spring Festival greeters as the social network size indicator (Bian, 2004, 2012). As the most important traditional festival in China, the Spring Festival is a common time for main social relations (such as relatives, friends, neighbors, and so on) to visit and greet each other. Over the last two decades, the Spring Festival greeting network has become an important measure of the social network or social capital of Chinese people (Bian, 2004, 2012). In the current study, participants were asked about the number of face-to-face interactions during the Spring Festival with three types of people: (1) relatives; (2) close friends; and (3) common friends, acquaintances, and other people. There were eight response categories ranging from *1* = *no one* to *8* = *more than 50* (for details, see Table 2). Even though these three items represent different aspects of social ties, they were strongly correlated, and the internal consistency was 0.70. This means that participants with more links with relatives often have more links with other social relations during the Spring Festival. Thus, in this study, scores of these three items were considered as measure indicators of social network size.

**TABLE 2:** Distribution of size of Spring Festival greeting network (*N* = 1068).

**Size**	**Relatives**	**Close friends**	**Common friends and other relationships**
			
	***n* (valid%)**	***n* (valid%)**	***n* (valid%)**
(1) 0	4 (0.4)	19 (1.8)	46 (4.3)
(2) 1∼2;	42 (3.9)	83 (7.8)	65 (6.1)
(3) 3∼4;	191 (17.9)	183 (17.2)	146 (13.7)
(4) 5∼10	370 (34.7)	357 (33.5)	312 (29.3)
(5) 11∼20	280 (26.2)	253 (23.8)	235 (22.1)
(6) 21∼30	96 (9.0)	105 (9.9)	120 (11.3)
(7) 31∼50	51 (4.8)	28 (2.6)	66 (6.2)
(8) More than 50	33 (3.1)	37 (3.5)	75 (7.0)
Missing	1	3	3

#### Future Time Perspective Scale (FTPS)

The 10-item FTPS consists of two sub-dimensions: seven items focus on future opportunities (FTP-opportunity) and three on the limitation of time (FTP-limitation) (Lang and Carstensen, 2002; Cate and John, 2007). Sample items of the two sub-dimensions included: “Most of my life still lies ahead of me” and “As I get older, I begin to experience time as limited,” respectively. Participants rated each item on a five-point Likert scale ranging from 1 (*strongly disagree*) to 5 (*strongly agree*). The Chinese version of the FTP demonstrated good internal consistency and construct validity (Zhang et al., 2019). In the present sample, Cronbach’s alpha coefficients for the two sub-dimensions were 0.85 and 0.70, respectively. Consistent with previous studies, a confirmatory factor analysis (CFA) indicated that the two-dimensional model of the data was significantly better than the unidimensional model (Cate and John, 2007). For the two-dimensional model: χ^2^(34) = 222.589, χ^2^/34 = 6.547, *p* < 0.001, CFI = 0.941, TLI = 0.922, RMSEA = 0.070 (90% CI: 0.061, 0.080), SRMR = 0.047. For the one-dimensional model: χ^2^(35) = 626.950, χ^2^/35 = 17.913, *p* < 0.001, CFI = 0.814, TLI = 0.761, RMSEA = 0.126 (90% CI: 0.117, 0.135), SRMR = 0.090. In addition, the factor loadings of all items in each sub-dimension were above 0.60. Thus, all items measure the respective construct they were designed to measure, indicating good internal consistency and construct validity. Given the above outcomes of the CFA and weak correlation between the two dimensions (*r* = −0.14, *p* < 0.01), we used them as two independent components of FTP in the analyses.

#### The Satisfaction With Life Scale (SWLS)

The SWLS comprises five items and is one of the most frequently used instruments to measure the cognitive aspect of SWB (Diener et al., 1985). The items are rated on a five-point scale ranging from 1 (*strongly disagree*) to 5 (*strongly agree*) (e.g., “*I am satisfied with my life*”). The Chinese version of the SWLS demonstrated good reliability, validity, and invariance across the sexes (Bai et al., 2011). Cronbach’s alpha coefficient was 0.85 in the present sample.

#### Positive Affect and Negative Affect Scale (PANAS)

PANAS comprises two subscales that describe one’s positive and negative moods (Watson et al., 1988). Using a five-point scale, participants indicated the extent of their feelings of positive affect (PA) (e.g., *excited* and *enthusiastic*) and negative affect (NA) (e.g., *distressed* and *upset*) during the past month. The Chinese version of PANAS has been confirmed as a reliable and valid instrument for assessing emotional state (Liang and Zhu, 2015). In this study, a short version was used to assess participants’ moods, and each subscale consisted of five items (Kercher, 1992). In the present sample, Cronbach’s alpha coefficient was 0.90 for the positive affect subscale and 0.78 for the negative affect subscale.

#### Meaning in Life Scale (MILS)

Meaning in life emphasizes the importance of seeking a worthy and meaningful life (McMahan and Estes, 2011), which represents the eudaimonic aspect of SWB. The eight-item MILS was designed to assess the sense of meaning and purpose in life (e.g., “*I have a philosophy of life that helps me understand who I am*”) (Krause, 2004). Participants rated each item on a five-point scale ranging from 1 (*strongly disagree*) to 5 (*strongly agree*). A higher aggregate score denotes a greater sense of meaning in one’s life. The reliability and structure of the Chinese version has been confirmed (Zhang and Zhang, 2017). In this study, Cronbach’s alpha coefficient was 0.85.

#### Covariates

The main demographic and sociological variables included age, sex, education level (elementary school or less, junior middle school, senior high school, junior college or higher), marital status (married [with a spouse] or single [separated, divorced, widowed, or never married]), and yearly household income (6 economic groups ranging from “less than RMB 20,000” to “more than RMB 120,000) (see Table 1). The self-rated yearly household income score was treated as a continuous variable in the analysis.

### Statistical Analysis

SPSS 22.0 was used to examine the descriptive statistics and. Mplus 7.0 (Muthén and Muthén, 2012) and the INDIRECT procedure (Preacher and Hayes, 2008) were employed to test the mediation model and mediation effects. The number of Spring Festival greeters with the three indicators was hypothesized as the predictor; FTP-opportunity and FTP-limitation as two mediating variables; SWB as a latent outcome variable represented by life satisfaction, negative affect, positive affect, and meaning in life; and demographic and socio-economic factors as covariates during the correlation and mediation analysis processes.

Based on previous studies, several indices were employed to evaluate the goodness-of-fit of the measurement and structural models on the mediating role (Bagozzi and Yi, 2012). These indices included the Chi square statistic value (χ^2^) and freedom degree, CFI (the Comparative Fit Index), TLI (the Tucker-Lewis-Index), RMSEA (the Root Mean Square Error of Approximation), and SRMR (standardized root mean square residual). According to Marsh et al. (2004), the following standards are recommended as the reference standards for a model’s acceptable fit: CFI ≥ 0.93, TLI ≥ 0.92, RMSEA ≤ 0.07, and SMRS ≤ 0.07.

### Results

#### Descriptive Findings

According to the distribution of the size of Spring Festival greeting networks, approximately one-third of participants reported a greeting network of 5–10 persons, and approximately 50–60% a greeting network of 5–20 persons for all three types of social relationships (see Table 2). This result is comparable to a previous survey (Bian, 2004), which found that the average size of the Spring Festival greeting network was approximately 28 people.

#### Measurement Model Test and Correlation Analyses

An initial test of the measurement model revealed that four latent (social network size, FTP-opportunity, FTP-limitation, and SWB) and 17 observed variables demonstrated an acceptable fit to the data: χ^2^(113) = 383.682, χ^2^/113 = 3.395, CFI = 0.949, TLI = 0.936, RMESA = 0.048 (90% CI: 0.043, 0.054), SRMR = 0.038. The CFA showed that the chosen manifest variables or items measured the respective latent construct they were designed to measure. All estimates were significant, indicating good internal consistency. Correlations of latent constructs that take into account for measurement errors indicated social network size was significantly related to FTP-opportunity, but not to FTP-limitation; overall, FTP-opportunity but not FTP-limitation was significantly related to SWB (see Table 3). It means that the mediating role of FTP likely comes from focusing on opportunities in the future, rather than on the limitation of time.

**TABLE 3:** Latent correlations based on Spring Festival greeting network.

	**1**	**2**	**3**
(1) Social network size	1		
(2) FTP_1	0.27***	1	
(3) FTP_2	0.04	–0.20***	1
(4) SWB	27***	0.49***	−0.06

FTP_1 and FTP_2 represent FTP-opportunity and FTP-limitation, respectively. ***p < 0.001.

#### Structural Model Test

Mplus 7.0 was employed to test the structural model of mediating roles using the maximum likelihood estimation. We tested whether FTP-opportunity and FTP-limitation mediate the relationship between social network size and SWB. In this model, FTP-opportunity and FTP-limitation were considered two independent mediators. First, according to the outcomes of the Mplus procedures, the goodness-of-fit structural model of mediation was not so satisfactory: χ^2^(183) = 676.034, χ^2^/df = 3.694, CFI = 0.905, TLI = 0.885, RMSEA = 0.052 (90% CI: 0.048, 0.056), SRMR = 0.042. Second, we found weak or insignificant correlations between FTP-limitation as a mediator, social network size as an independent variable, and the four indicators of SWB as dependent variables. There was no significant mediating effect among the aforementioned three variables. Thus, we conducted a single-mediation analysis with only FTP-opportunity as the mediator. A retest of the measurement model indicated that the three latent variables with 14 observed variables demonstrated a satisfactory fit to the data: χ^2^(73) = 267.691, χ^2^/113 = 2.369, CFI = 0.959, TLI = 0.949, RMSEA = 0.050 (90% CI: 0.044, 0.056), SRMR = 0.034. The structural model test revealed that the single-mediator model was better than the two-mediator model and the goodness-of-fit was acceptable: χ^2^ (130) = 419.130, χ^2^/df = 3.224, CFI = 0.937, TLI = 0.922, RMESA = 0.047 (90% CI: 0.042, 0.052), SRMR = 0.037.

#### Mediation Effect

**FIGURE 1:**
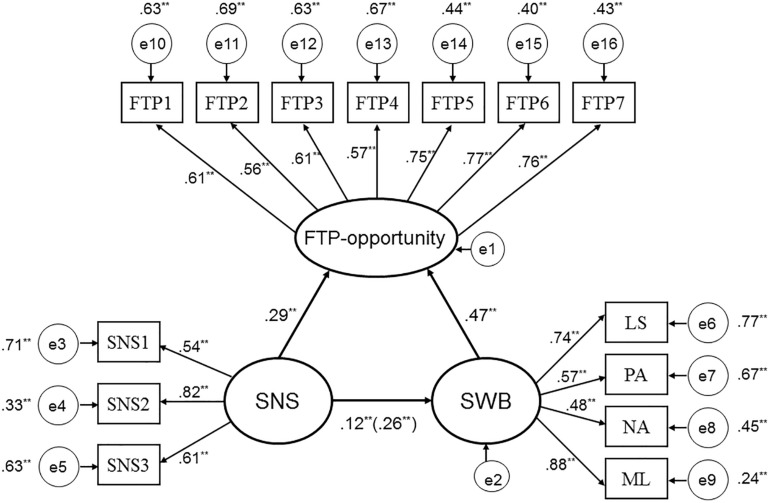
The structural model on mediating role of FTP-opportunity (*N* = 1068). Factor loadings and path coefficients are standardized; SNS represents social network size; SNS1, SNS2, and SNS3 represent sizes of three types of Spring Festival greeting network, respectively; LS, PA, NA, and ML, represent life satisfaction, positive affect, negative affect, and meaning in life, respectively. Roles of demographic and socio-economic variables in SWB and FTP-opportunity were controlled for.

According to the standardization outcome of the mediating analysis, the mediating role of FTP-opportunity was significant, and the proportion of mediating effect in total effect was 0.135/0.256 = 0.527. After controlling for the indirect effect of FTP-opportunity, the direct effect of social network size on SWB remained significant (Estimate = 0.120, *p* < 0.01). This indicates that FTP-opportunity played a semi-mediating role. The results of the INDIRECT procedures using 5,000 bootstrapped samples indicated that the 95% bias-corrected confidence interval of the indirect effect did not contain zero (0.095, 0.174), confirming the mediated effect of FTP-opportunity as significant (see Figure 1).

#### Roles of Demographic and Socio-Economic Variables

The main outcome of this study was demonstrating the relationships between social network, FTP, and SWB. However, the roles of some demographic and socio-economic factors are also noteworthy. Household income (β = 0.232, *p* < 0.001), education (β = 0.078, *p* < 0.05), and age (β = 0.065, *p* < 0.05) were associated with SWB. That is, retirees with a higher household income, education level, and age have more opportunities to experience SWB. In addition, a higher age was related to higher SWB and lower FTP-opportunity.

## Study 2: Size of Networks Based on Common Actions as the Independent Variable

In Study 1, the number of Spring Festival greeters only represents the scale of social interaction in this cultural context. In Study 2, we used the “size of networks based on common actions” as the indicator of social network size. These types of social networks (e.g., discussion networks, mutual helping networks, and common participation networks) can broadly reflect social interactions, are more adaptable to diverse cultural contexts, and have been applied in studying the social relationships of Chinese people (Ruan et al., 1997; Liu, 2006; Bian, 2012). Using core discussion network, Ruan et al. (1997) examined the structure of social network and its change with time in Urban China. Liu (2006) investigated the mutual support network in a village of China. This type of social network based on mutual help or support was considered a very beneficial method to study the Chinese society where *guanxi* (relationship) is particularly emphasized.

### Participants and Procedure

Using a similar sampling method, 355 retirees aged 50 years or older from 11 neighborhoods in same city with Study 1 were selected as participants, and 335 validly completed the questionnaires (valid response rate = 94%) (see Table 4 on demographic and socio-economic variables). In Study 2, social network size was based on common actions. All other variables, instruments, methods of analysis, and procedures were the same as those in Study 1. The investigation was conducted from June to August 2017. Each participant took approximately 30 min to complete this investigation. Once completed, each participant was paid RMB 40 (about $6) as compensation.

**TABLE 4:** Demographic and socio-economic characteristics of participants in Study 2.

**Variable**	***n***	**valid%**	**Mean(SD)**
**Sex**			
Male	146	43.7	
Female	188	56.3	
Missing	1		
**Age**			63.12 (8.02)
59 and younger	108	32.4	
60–69 years	160	48.0	
70–79 years	51	15.3	
80 and older	14	4.2	
Missing	2		
**Educational level**			
Elementary or lower	50	15.5	
Junior high school	150	46.4	
Senior high school	91	28.2	
Junior college or higher	32	9.9	
Missing	12		
**Marital status**			
Single(widowed/unmarried/divorced/separated)	46	14.2	
With a spouse	277	85.8	
Missing	12		
**Household**			
**Household yearly income(RMB)**			
Less than 20,000	60	18.4	
20,000-30,000	87	26.7	
30,000–50,000	110	33.7	
50,000–80,000	48	14.7	
80,000–120,000	19	5.8	
More than 120,000	2	0.6	
Missing	9		

### Measures

In Study 2, we used three items to assess the size of the retirees’ social network. Participants were asked, looking back the past year: (1) “How many people did you once discuss important issues with whether in life, work, or other fields?” (2) “How many people did you once offer important help to for each other in life, work, or other fields?” (3) “With how many people did you once participate in important social activities within life, work, or other fields?” The seven response categories ranged from *1* = *no one* to *7* = *no less than 15 people*. Previous studies highlight that discussion, mutual helping, and participating in common activities are important aspects of social networks and reflect a broad and general social relationship (Ruan et al., 1997; Liu, 2006; Bian, 2012). The three items were strongly correlated. The internal consistency was 0.84 and pairwise correlations were between 0.58 and 0.70. In this study, these three items were considered measure indicators of social network size.

### Statistical Analysis

As in Study 1, SPSS 22.0 was used to examine the descriptive statistics. Mplus 7.0 (Muthén and Muthén, 2012) and the INDIRECT procedure (Preacher and Hayes, 2008) were employed to test the mediation effects. The size of the social network was hypothesized as the predictor, FTP-opportunity and FTP-limitation as two mediating variables, SWB was represented by the four indicators as the dependent variables and demographic and socio-economic factors as covariates during the mediation analysis processes.

### Results

#### Descriptive Findings

According to the distribution of the size of social networks based on common actions, approximately 60–70% of participants reported that the sizes of their discussion network, mutual helping network, and common participation network were three to eight persons (see Table 5). This result is consistent with that of previous surveys (Burt, 1984; Bian, 2012).

**TABLE 5:** Distribution of size of networks based on common actions (*N* = 335).

	**Discussion**	**Helping**	**Participation**
			
**Size**	***n* (valid%)**	***n* (valid%)**	***n* (valid%)**
(1) 0	1 (0.3)	4 (1.2)	2 (0.6)
(2) 1	14 (4.2)	6 (1.8)	11 (3.3)
(3) 2	56 (16.8)	33 (9.9)	18 (5.4)
(4) 3∼4	149 (44.7)	119 (35.6)	97 (29.1)
(5) 5∼8	84 (25.2)	108 (32.3)	118 (35.4)
(6) 9∼14	16 (4.8)	33 (9.9)	51 (15.3)
(7) 15∼	13 (3.9)	31 (9.3)	36 (10.8)
Missing	2	1	2

#### Measurement Model Test and Correlation Analyses

The measurement model test revealed that four latent variables (social network size, FTP-opportunity, FTP-limitation, and SWB) and 17 observed variables demonstrated an acceptable fit to the data: χ^2^(113) = 221.385, χ^2^/113 = 1.959; CFI = 0.946; TLI = 0.933; RMSEA = 0.055 (90% CI: 0.044, 0.065); SRMR = 0.056. The CFA showed that the chosen manifest variables measured the respective latent construct they were designed to measure, and all estimates were significant, indicating good internal consistency. Consistent with the outcome of Study 1, correlations of latent constructs indicated that social network size was significantly related to FTP-opportunity, but not to FTP-limitation; overall, FTP-opportunity but not FTP-limitation was significantly related to SWB (see Table 6). It means that the mediating role of FTP likely comes from focusing on opportunities in the future, rather than on the limitation of time.

**TABLE 6:** Latent correlations based on common actions network.

	**1**	**2**	**3**
(1) Social network size	1		
(2) FTP_1	0.20***	1	
(3) FTP_2	0.08	0.19^**^	1
(4) SWB	0.27***	0.70***	−0.06

FTP_1 and FTP_2 represent FTP-opportunity and FTP-limitation, respectively. ^**^p < 0.01; ***p < 0.001.

#### Structural Model Test

First, according to the outcomes of Mplus procedures, goodness-of-fit of the mediation model with two mediators was not satisfactory: χ^2^(183) = 676.034, χ^2^/df = 3.694; CFI = 0.908; TLI = 0.888; RMESA = 0.058 (90% CI: 0.050, 0.067); SRMR = 0.058. Consistent with the outcomes of Study 1, there were weak or insignificant correlations between FTP-limitation and social network size and SWB. Thus, we conducted a mediating analysis with only FTP-opportunity as the mediator. First, the retest of the measurement model indicated that three latent variables with 14 observed variables demonstrated a satisfactory fit to the data: χ^2^(74) = 126.122, χ^2^/113 = 1.752; CFI = 0.970; TLI = 0.962; RMSEA = 0.047 (90% CI: 0.033, 0.061); SRMR = 0.042. The structural model test revealed that the single-mediator model was better than the two-mediator model and the goodness-of-fit was satisfactory: χ^2^(134) = 235.443, χ^2^/df = 1.757; CFI = 0.941; TLI = 0.928; RMSEA = 0.051 (90% CI: 0.040, 0.061); SRMR = 0.044.

#### Mediation Effect

**FIGURE 2:**
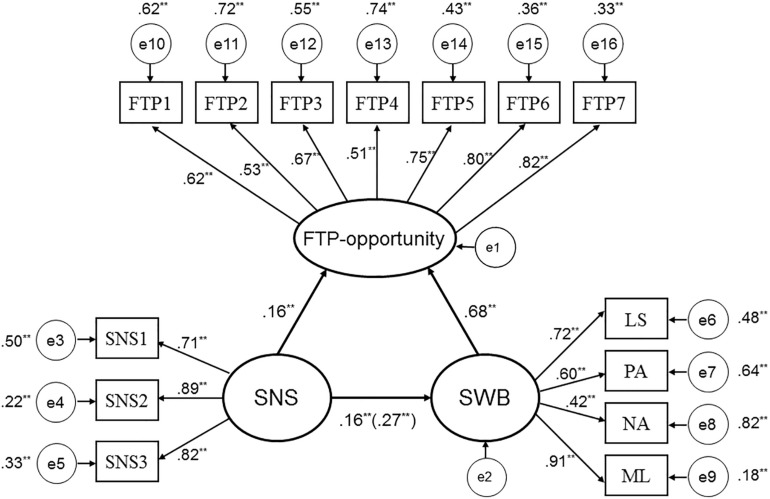
The structural model on mediating role of FTP-opportunity (*N* = 335). Factor loadings and path coefficients are standardized; SNS represents social network size; SNS1, SNS2, SNS3 represent sizes of three network relationships, respectively: LS, PA, NA, and ML, represent life satisfaction, positive affect, negative affect, and meaning in life, respectively. Roles of demographic and socio-economic variables in SWB and FTP-opportunity were controlled for.

According to the standardization outcome of the mediating analysis, the mediating role of FTP-opportunity was significant; and the proportion of mediating effect in total effect was 0.110/0.270 = 0.407. After controlling for FTP-opportunity, the direct effect of social network size on SWB remained significant (Estimate = 0.160, *p* < 0.01), indicating that FTP-opportunity played a semi-mediating role. The INDIRECT indicated that the 95% bias-corrected confidence interval of the indirect effect did not contain zero (0.026, 0.195), showing that the mediating effect of FTP-opportunity was significant (see Figure 2).

#### Roles of Demographic and Socio-Economic Variables

Given that Study 2 had a relatively smaller sample size, the effect of some demographic and socio-economic variables on SWB decreased or disappeared. Only yearly household income was positively associated with SWB (β = 0.171, *p* < 0.01).

## Discussion and Conclusion

### Social Network Size, FTP, and SWB

The two studies revealed that social network size was positively associated with SWB, which was indicated by life satisfaction, positive affect, negative affect, and meaning in life among retired people. Retirees with broader social ties were more likely to have higher life satisfaction, positive affect, sense of meaning, and weaker negative affect to varying degrees after adjusting for the role of demographic and socio-economic factors. First, consistent with influential studies (Pinquart and Sörensen, 2000), our research showed that social network size was related to both hedonic and eudaimonic aspects of SWB, and this positive role of social network size was further confirmed among middle-aged and older adults in China (Litwin and Shiovitz-Ezra, 2011; Moorman and Boerner, 2017). Second, the results revealed that both the size of the Spring Festival greeting network based on cultural background and networks based on common actions benefited the SWB of retired residents (Ma, 2015). The sizes of both types of social networks reflect the quantity and quality of one’s social relationship (so-called popularity), which are considered a direct source of SWB, confirming Hypothesis 1.

Both studies also confirmed the positive pairwise correlations among social network size, general FTP score, and SWB. However, specific analysis showed that only FTP-opportunity was positively associated with social network size and SWB. Social network size, whether the Spring Festival greeting network or networks based on common action, was positively associated with FTP and the sub-dimension FTP-opportunity, but not the sub-dimension FTP-limitation. Furthermore, FTP and FTP-opportunity, but not FTP-limitation, were positively associated with SWB. The correlation and mediating analyses showed that opportunity and limitation are relatively independent components that influence SWB differently. Researchers found that FTP-opportunity and FTP-limitation correlated differently with mental health and SWB (Cate and John, 2007; Kozik et al., 2015). Thus, Hypotheses 2 and 3 were partly confirmed.

### Mediating Role of FTP

Further mediating analyses indicated that FTP-opportunity, but not FTP-limitation, partly mediated the correlations between social network size and SWB. We infer that a large social network or the enlarging thereof can enhance hope regarding the possibility of achieving goals and plans, but not change one’s perception of the limitation of remaining time. Thus, Hypothesis 4 was partly confirmed.

First, we speculated that large and diverse social networks can enhance the social activities and communication of retired middle-aged and elderly people, subsequently leading them to view their future more positively. Positive views of the future may further enhance their happy experience of life. Second, according to social resource theory (House et al., 1988; Lin, 2001), the social network is a main channel for the transportation and exchange of various resources. Thus, large and diverse social networks endow individuals with more social and emotional resources, and create helpful conditions to implement tasks in the future. As the chronological age of participants was controlled in the analyses, we inferred that social networks subjectively extend perceptions regarding future plans, causing older adults to be more focused on future opportunities and less on current constraints. Researchers found that FTP is positively correlated with optimism and negatively correlated with pessimism (Allemand et al., 2012). Third, relative to restricted networks, large social networks provide an important source for acquiring new information and knowledge. Outside information is crucial for older adults who have retired. With large networks, they can acquire more new knowledge on healthcare and health-promoting measures (Shiovitz-Ezra and Litwin, 2012), which may enhance their expectations regarding future life. In addition, retirees with a bigger social network often have diverse social or family roles, and a higher chance of getting along with younger people. Consequently, they have a youthful outlook regarding future life. However, these inferences must be sufficiently supported by empirical evidence from future research.

### Limitations

Several limitations in the design of this study must be pointed out. First, it is impossible to infer causal relationships through a cross-sectional design. Logically, we hypothesized that network size affects perceptions regarding future time length and opportunities, which enhances or maintains SWB. However, the relationships between variables could go in other directions. Therefore, employing an experimental or longitudinal design in the future is recommended to explore the relevant causality and mediating mechanisms. Second, we focused on the role of the general size of the social network as the independent variable, which does not consider the effect of other features of a social network such as the composition or type of network, which are considered influential factors regarding a person’s psychology and behavior. For example, Huxhold et al. (2013) found that network type enhanced the social engagement and emotional support of older adults, indirectly influencing SWB. Future studies should consider using multidimensional indicators as a social network measure in future studies. Third, researchers should consider whether the Spring Festival greeting network or networks based on common actions accurately represent one’s relatively intimate relationships. For example, discussing important issues or mutual helping are limited to the scope of family, relatives, or close friends, and does not provide a complete view of a social network. With rapid development of Internet technology, social relationship with others only known over the computer and smartphone became increasingly popular, and now constitute an important part of social network of middle aged and elderly people, which provide a promising research field on psychological outcomes of social network (Wilson et al., 2012). Forth, the authors used four indicators of SWB, three related to hedonic well-being and only one related to eudaimonic well-being (meaning in life). However, eudaimonic well-being is also a multifacet construct (Ryff, 1989; Disabato et al., 2015). According to Ryff’s (1989) psychological well-being model, eudaimonia includes six factors: self-acceptance, positive relations with others, autonomy, environmental mastery, purpose in life, and personal growth. Future studies should use a more comprehensive measure to assess one’s eudaimonic well-being. In addition, China has the largest elderly population and retirees represent a relatively special sample of older adults. In China, retirees mainly consist of those people who once worked as regular staff in public institutions, enterprises, and government sectors. Compared with other elderly people (especially those who live in rural areas), retired city people as the body of the Chinese retired population often enjoy stable pensions, sufficient medical security, and various other welfares. Despite that, current findings at least in some ways reflect importance of social relations in maintaining SWB among elderly urban residents. Future studies can demonstrate above hypotheses or models from a broader representative population and consider cross-cultural comparison.

Despite these limitations, this study was an initial exploration to understand the relation between social network size and the SWB of middle-aged and older Chinese individuals. First, our findings added new evidence of the effects of enhancing and maintaining social relationship, especially the size or number of social network, on the positive psychology. Moreover, this study applied two approaches to assess network size: the Spring Festival greeting network with specific cultural characteristics, and networks based on common actions adaptable to cross-cultural contexts, which increases the generalizability of the findings. Second, and most important, our findings partially attribute the positive effect of social networks on SWB to FTP, adding new knowledge concerning the health-enhancing effects of social relationships. Individuals can improve their SWB by expanding their social relationships and cultivating a positive and open-ended sense of the future. The findings of this study provide helpful information for local governments and communities in terms of management and services as well as in the creation and implementation of relevant policies.

## Data Availability Statement

The raw data supporting the conclusions of this manuscript will be made available by the authors, without undue reservation, to any qualified researcher.

## Ethics Statement

The academic and ethics committees of Institute of Psychology, Chinese Academy of Sciences approved this study. After the investigators explained the aim and requirements, all participants provided written informed consent during conducting the investigation. Participation in the study was anonymous. The study was conducted in full accordance with the Ethical Guidelines of the American Psychological Association (APA).

## Author Contributions

ZZ conceived the study, conducted the analyses, and drafted the manuscript. ZZ and JZ contributed to the conceptualization, measurement, and analyses. ZZ, NZ, and YY performed the measurement and contributed to the analyses. All authors read, modified, and approved the final manuscript.

## Conflict of Interest

The authors declare that the research was conducted in the absence of any commercial or financial relationships that could be construed as a potential conflict of interest.
